# Low-Effort Respiratory Function Estimation with a Soft Wearable Digital Spirometry Patch

**DOI:** 10.3390/bios16050272

**Published:** 2026-05-08

**Authors:** Faheem A. Karim, Ahmed Tariq, Christopher B. Fitzpatrick, Lauren Zhou, Mayte Suárez-Fariñas, Helena Schotland, Linda Rogers, Yoon Jae Lee, Woon-Hong Yeo, Yun Soung Kim

**Affiliations:** 1 Icahn School of Medicine at Mount Sinai, New York, NY 10029, USA; 2 BioMedical Engineering and Imaging Institute, Icahn School of Medicine at Mount Sinai, New York, NY 10029, USA; 3 School of Electrical and Computer Engineering, Georgia Institute of Technology, Atlanta, GA 30332, USA; 4 George W. Woodruff School of Mechanical Engineering, Georgia Institute of Technology, Atlanta, GA 30332, USA; 5 Center for Biostatistics, Department of Population Health Science and Policy, Icahn School of Medicine at Mount Sinai, New York, NY 10029, USA; 6 Icahn Institute for Data Science and Genomic Technology, Icahn School of Medicine at Mount Sinai, New York, NY 10029, USA; 7 Department of Computer Science, Georgia State University, Atlanta, GA 30303, USA; 8 Wallace H. Coulter Department of Biomedical Engineering, Georgia Tech and Emory University, Atlanta, GA 30332, USA; 9 Wearable Intelligent Systems and Healthcare Center (WISH Center), Institute for Matter and Systems, Georgia Institute of Technology, Atlanta, GA 30332, USA; 10 Parker H. Petit Institute for Bioengineering and Biosciences, Georgia Institute of Technology, Atlanta, GA 30332, USA; 11 Department of Diagnostic, Molecular, and Interventional Radiology, Icahn School of Medicine at Mount Sinai, New York, NY 10029, USA

**Keywords:** wearable biosensor, spirometry, FVC, FEV_1_, PEF, linear regression, elastic net, feature extraction, cross-validation

## Abstract

Spirometry is widely regarded as the clinical gold standard for quantifying lung function. It plays a central role in the diagnosis and management of cardiopulmonary disorders, including asthma and chronic obstructive pulmonary disease (COPD). However, the procedure relies on a forceful and often stressful expiratory maneuver that may cause patient discomfort and require substantial effort, frequently necessitating active coaching and trained personnel to ensure reproducible measurements. In this paper, we present the Digital Spirometry Patch (DSP), a soft, flexible, wearable patch capable of estimating lung function parameters by utilizing low-effort breathing maneuvers. Eighteen participants performed low-effort and forceful breathing maneuvers while wearing the DSP to collect tracheal sound and chest movement signals for spirometric parameter estimation using elastic net and simple linear regression. Using leave-one-subject-out cross-validation, the elastic net models achieved RMSEs of 0.668 L, 0.224 L, and 0.428 L/s for FVC, FEV_1_, and PEF, respectively, using low-effort breathing maneuvers, and 0.499 L, 0.304 L, and 0.891 L/s using forceful exhalation maneuvers. These results demonstrate the potential of the DSP as a wearable, low-effort alternative for estimating lung function outside of conventional spirometry settings.

## 1. Introduction

Chronic respiratory diseases such as asthma, chronic obstructive pulmonary disease (COPD), interstitial lung diseases (ILDs), and pulmonary sarcoidosis affect millions globally and contribute significantly to long-term morbidity [1]. Timely and accurate diagnosis is essential for effective respiratory disease control. Among the available diagnostic modalities, spirometry is widely regarded as an important tool for the diagnosis and management of respiratory disorders such as asthma and COPD [2]. Spirometry quantifies airflow and basic lung volumes such as vital capacity, providing an objective measure of pulmonary function. A standard spirometry procedure involves a patient taking a deep inhalation to reach total lung capacity, followed by a rapid, continuous, and forceful exhalation into a spirometer through a mouthpiece or tube. During the exhalation maneuver, patients are required to wear a nose clip and maintain a tight seal around the mouthpiece to prevent air leakage and ensure accurate measurement. The patient will then repeat this sequence until three consistent trials are recorded [3]. The spirometry test returns several baseline lung function parameters, such as forced vital capacity (FVC), forced expiratory volume in one second (FEV_1_), FEV_1_/FVC ratio (FEV_1_%), and peak expiratory flow (PEF). Collectively, these metrics enable clinicians to characterize the mechanical properties of the lungs, monitor disease progression, and evaluate the impact of therapies.

Each step of spirometry depends heavily on patient effort and cooperation, so trained respiratory personnel are typically needed to ensure consistent, reliable measurements across repeated trials. During testing, technicians must provide real-time coaching to elicit maximal inspiratory and expiratory effort, verify proper seal and posture, identify suboptimal maneuvers, and judge whether reproducibility criteria defined by standardized guidelines have been satisfied. These demands can make spirometry burdensome and uncomfortable, particularly for older or debilitated patients, increasing the likelihood of inaccurate results [4,5]. In addition, conventional spirometry requires routine calibration, ongoing maintenance, and a controlled testing environment, conditions that are often difficult to reproduce outside of clinical settings and may not be available in resource-limited regions. Diagnostic errors are further exacerbated by limited access to spirometry in primary care, where many asthma cases are first evaluated, thereby increasing the risks of misdiagnosis, underdiagnosis, and overdiagnosis [6]. Such errors can have important clinical consequences. For example, treating asthma as COPD, despite overlapping symptoms, may delay appropriate therapy and contribute to irreversible lung damage [7]. Conversely, over-treatment of asthma with oral corticosteroids can produce systemic adverse effects, including musculoskeletal, psychiatric, cardiovascular, ocular, and metabolic complications [8], whereas under-treatment may lead to recurrent exacerbations, progressive airway remodeling [9], and death [10].

Recently, several portable and lightweight systems have been developed to support respiratory assessment outside of the clinic, ranging from handheld spirometers to smartphone-based tools and wearable respiratory monitors. Handheld spirometers, such as MIR’s Spirobank Smart [11], remain the most established option for at-home lung function testing and can generate standard spirometric parameters, including FVC, FEV_1_, and PEF. However, like conventional spirometry performed in clinical settings, these devices still depend on maximal forced expiratory maneuvers and often require additional accessories, such as a mouthpiece and a nose clip, which can limit convenience and contribute to improper use in unsupervised settings. Studies have shown that limited user guidance and insufficient familiarity with spirometry during at-home testing can lead to poor agreement with clinically obtained measurements [12,13].

Beyond handheld spirometers, several groups have explored smartphone-based approaches as lower-cost and more accessible alternatives for estimating lung function. Studies from the University of Washington, including SpiroCall [14] and SpiroSmart [15], demonstrated that exhalation sounds captured with a smartphone microphone can be used to estimate spirometric indices, whereas SpiroSonic [16] further incorporated sensing of chest wall motion alongside exhalation sound. Other related studies used alternative surrogate maneuvers and sensing configurations, such as cough sounds recorded with a smartphone microphone [17] or speech sounds recorded using an external microphone [18], to predict selected pulmonary parameters. These studies support the feasibility of extracting lung function information from audio-based signals, but they still rely on maneuvers that mimic key aspects of forced spirometry and remain susceptible to practical confounders, such as user positioning, signal quality, variability in sound acquisition, and limited ability to distinguish inspiratory from expiratory phases [16,17,19]. In addition, these approaches generally provide single time-point assessments rather than continuous or longitudinal respiratory monitoring.

Several wearable systems have also been developed to enable passive respiratory monitoring over extended periods. For example, chest-worn platforms such as Resmetrix have been designed for normal breathing assessment of respiratory patterns [20], whereas other wearable chest-mounted devices, including the Strados Lab Resp^®^ Biosensor [21] and ADAMM-RSM-SM [22], support continuous monitoring of chest sounds and motion for detection of adventitious respiratory events, such as wheezes, rhonchi, and crackles. Our earlier soft wearable stethoscope patch similarly demonstrated the utility of a skin-conformal patch for wearable respiratory sound monitoring and adventitious sound detection [23,24]. Although these wearable systems are valuable for passive and longitudinal respiratory surveillance, they do not generate spirometry-equivalent parameters and therefore do not directly provide objective measures of lung function, such as FVC, FEV_1_, and PEF.

Collectively, existing approaches highlight a tradeoff between direct spirometric quantification and ease of use. Handheld spirometers can generate standard lung function parameters but still require forceful expiratory effort and external accessories. Smartphone-based systems reduce hardware burden but remain sensitive to user behavior, recording conditions, and maneuver variability. Wearable respiratory monitors enable low-burden longitudinal sensing yet generally do not output standard spirometric metrics. To our knowledge, no prior system has simultaneously combined a low-effort maneuver, generation of standard spirometric parameters, and a fully self-contained wearable form without additional accessories.

**Figure 1:**
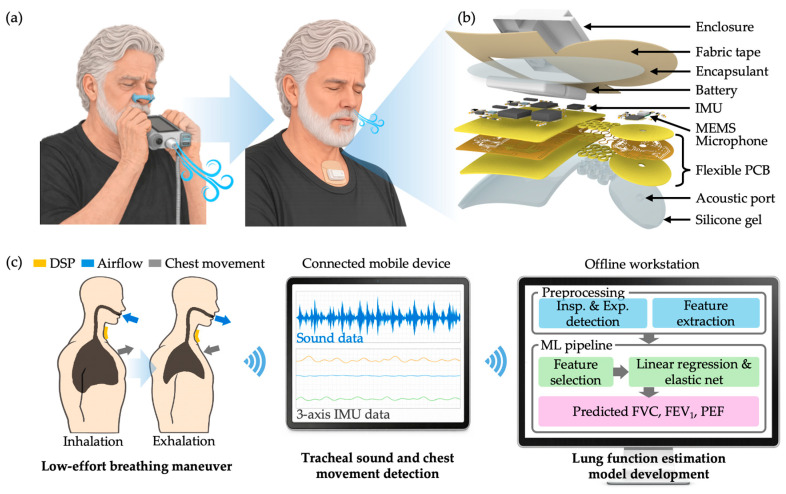
Overview of the Digital Spirometry Patch (DSP) project. (**a**) Illustrative comparison of respiratory maneuvers used in conventional spirometry and DSP. Conventional spirometry requires forceful user effort, whereas DSP is designed to operate with lower user effort, enabling easier and more frequent use in remote settings while still generating clinically relevant lung function parameters. (**b**) Exploded view of the DSP, highlighting the substrate, MEMS sensor, conductive traces, acoustic coupling interface, and integrated power system. (**c**) Data flow and processing. (**Left**) Illustrative cross-sectional views showing how the DSP captures tracheal sound and chest movement during the exhalation maneuver. (**Center**) Real-time sensor data are displayed and recorded on a Bluetooth-connected tablet. (**Right**) Flowchart of the offline data processing pipeline used to generate the predicted lung function parameters (FVC, FEV1, PEF).

Here, we present an all-in-one, non-invasive wearable device, termed the Digital Spirometry Patch (DSP), designed to address these limitations and distinguished from prior approaches by its ability to support low-effort lung function assessment, spirometry-equivalent output, and a self-contained wearable form factor (Table 1). Unlike conventional spirometry, which depends heavily on patient effort, technician supervision, and controlled testing conditions, the DSP is designed to support lung function assessment through a simpler breathing maneuver in which the user takes a deep breath and exhales gently, without the need for intensive coaching, specialized facilities, or external accessories (Figure 1a). Building on our previous patch-based platform for detecting adventitious respiratory sounds such as wheezes, crackles, and rhonchi [23,24], the DSP extends this wearable, body-conformal format toward quantitative lung function assessment through integrated acoustic and inertial sensing in a compact patch (Figure 1b). Specifically, the DSP captures tracheal airway sounds together with chest wall motions from an inertial measurement unit (IMU) across the breathing maneuver, providing complementary information related to respiratory airflow and thoracic motion. These multimodal signals are displayed and recorded on a connected mobile device and processed offline to predict reference spirometric parameters, including FVC, FEV_1_, and PEF, conventionally obtained through standard forced-expiration spirometry (Figure 1c). To evaluate this approach, participants performed breathing maneuvers while wearing the DSP alongside standard spirometry, enabling direct comparison between patch-derived predictions and reference measurements. By framing lung function assessment in a wearable, low-burden format, this study explores a path toward more accessible and more frequent pulmonary monitoring in remote and home-based settings.

## 2. Materials and Methods

### 2.1. Ethics Statement

This study was conducted at the BioMedical Engineering and Imaging Institute, Icahn School of Medicine at Mount Sinai, New York, NY. Ethical approval was obtained from the Institutional Review Board (IRB; STUDY-23-01211), and all participants provided written informed consent prior to enrollment. The trial was conducted in accordance with International Council on Harmonisation Good Clinical Practice (ICH GCP) guidelines.

### 2.2. Study Design

This was a single-site, single-arm, observational validation study employing a within-subjects repeated measures design. Each participant served as their own control, with spirometry values obtained via a clinical-grade spirometer (EasyOne Air, manufactured by Ndd Medical Technologies, Andover, MA, USA) used as the reference standard for algorithm validation. The primary endpoints were the root mean square error (RMSE) and mean absolute error (MAE) between model-predicted and spirometry-measured values for FVC, FEV_1_, and PEF, calculated from held-out breath predictions obtained via leave-one-subject-out cross-validation (LOSO-CV).

### 2.3. Sample Characteristics

Demographic characteristics of the eighteen healthy participants can be found in Table 2.

### 2.4. Device Description

The Digital Spirometry Patch (DSP) is a skin-conformal, wireless patch that simultaneously acquires tracheal sounds and chest motion data. The device integrates a high-fidelity microphone (ICS-40212, TDK InvenSense, San Jose, CA, USA) for acoustic capture and inertial measurement units (IMUs; ADXL355, Analog Devices, Wilmington, MA, USA) for chest kinematic sensing. A block diagram describing the key electronic components used in the DSP, as well as downstream sensor data utilization, is shown in Figure S1 in the Supplementary Information. The patch was applied to a standardized anatomical location—anteriorly over the first and second intercostal spaces (conforming over the suprasternal notch) using a non-invasive, medical-grade adhesive backing (Silbione RT Gel 4717, Bluestar Silicones, East Brunswick, NJ, USA). Correct placement and secure skin contact were confirmed by a trained research staff member prior to initiating any breathing maneuvers. No external accessories, such as mouthpieces or nose clips, were required during patch-only recording conditions.

### 2.5. Randomization

To reduce order-related bias, each participant was assigned to a predetermined randomized sequence of maneuver types prior to enrollment. Sequences were generated using a computerized random number generator in R (version 4.3.1) with a fixed seed to ensure reproducibility, and participants were allocated equally across available maneuver ordering permutations by the study biostatistician. Although participants were informed that they would complete a series of respiratory tasks, the specific maneuver type and order were disclosed only immediately before each task, minimizing anticipatory behavioral adjustment. Research staff responsible for guiding participants were not blinded due to the real-time instruction requirements of the protocol; however, data analysts were blinded to participant identifiers throughout model training and evaluation.

### 2.6. Study Procedure

All procedures were completed during a single in-person visit lasting approximately one to two hours. Upon arrival, height and weight were measured using a calibrated instrument (500KL, Health o meter Professional Scales, McCook, IL, USA) to compute BMI, while age, sex, and race/ethnicity were self-reported via a brief questionnaire. After baseline measurements were obtained, the patch was applied and participants completed a randomized sequence of guided respiratory maneuvers organized into three categories: (1) traditional spirometry, in which participants used the handheld spirometer to inhale deeply and exhale forcefully (maximum of 8 repetitions) per the coaching standards recommended by the American Thoracic Society (ATS); (2) low-effort breathing, consisting of slow, deep inhalation followed by nasal exhalation, resembling the breathing pattern used during routine auscultatory lung assessment; and (3) forceful breathing, consisting of a deep inhalation followed by a sharp, rapid mouth exhalation without the spirometer device, similar in effort level to conventional spirometry. At the conclusion of the session, participants were asked to fill out a survey assessing their experience using the DSP and their perspectives on future use. Although no formal statistical analysis was performed with the self-reported responses, overall feedback regarding patch usage and utility was positive. The summary of these findings is presented in Table S1 and Figure S2 in the Supplementary Information.

### 2.7. Respiratory Phase Segment Classification and Feature Extraction

**Figure 2:**
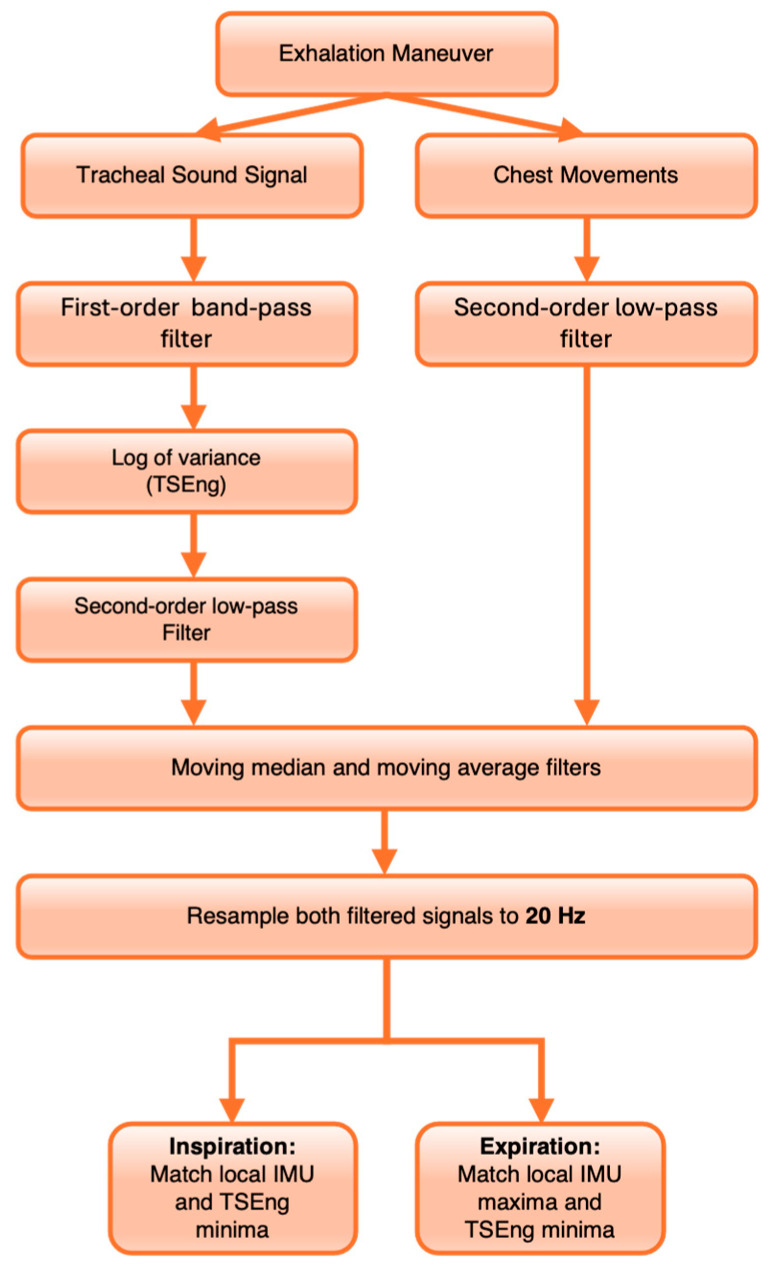
Overview of the respiratory phase detection pipeline.

Inspiration and expiration are characterized by distinct acoustic and thoracic motion properties, which necessitates their explicit identification as separate segments. Inspired by the work of Ghahjaverestan et al. [25], who developed a pipeline for estimating respiratory airflow and tidal volume from tracheal sound and motion signals, we adapted and tailored their breath detection pipeline for implementation in the DSP. Prior to feature extraction, tracheal sound and IMU accelerometer data were processed to identify respiratory phases, as summarized in Figure 2. For the IMU data, the Z-axis acceleration signal was extracted and low-pass filtered using a second-order Butterworth filter (cutoff frequency: 5 Hz) with zero-phase filtering to isolate respiration-related motion. Previous work has shown that chest motion alone can be used to detect breathing [26]; here, tracheal sound was incorporated as a complementary signal to account for artifacts in IMU-derived measurements. For the acoustic signal, the stethoscope recording was band-pass filtered between 50 and 1000 Hz to emphasize tracheal sounds. Tracheal sound energy (TSEng) was estimated using the logarithm of the moving variance of the filtered audio signal computed over a 20 ms sliding window. This signal was then subsequently low-pass filtered at 2 Hz to produce a smooth respiratory envelope.

To identify respiratory events, both the IMU signal and the TSEng envelope were further smoothed using moving median and moving average filters to suppress small fluctuations. The smoothed signals were resampled to 20 Hz to facilitate peak detection. Local extrema were then identified using a peak detection method based on prominence and distance criteria, with a minimum peak distance of 1.5 s and a prominence threshold defined as 20% of the signal interquartile range. Respiratory phase events were determined by associating extrema from the IMU signal with a nearby minima in the TSEng envelope within a ±1 s temporal window. IMU minima that aligned with TSEng minima were labeled as the start of inspiration events, whereas IMU maxima were labeled as the start of expiration events. The identified events were used to segment the recordings into inspiration and expiration intervals. To identify valid respiratory cycles, inspiration events were required to be followed sequentially by an expiration event and a subsequent inspiration event marking the end of expiration to be considered a valid segment pair.

For each inspiration and expiration segment within a valid breathing cycle, both acoustic and motion features were extracted, and 13 mel-frequency cepstral coefficients (MFCCs) were computed for each breath segment. MFCCs are features that summarize the overall shape of the spectral envelope of an audio signal and are commonly used in audio recognition tasks. They are obtained by applying a cosine transform to the logarithm of the short-term audio spectrum represented on a mel-frequency scale [27]. MFCCs have also been utilized in cardiopulmonary signal analysis in applications such as adventitious lung sound detection [28] and heart sound classification [29]. Given their ability to capture short-term spectral characteristics, we chose MFCCs as the primary audio features for modeling tracheal sounds generated during breathing maneuvers. The 13 MFCCs were calculated from the band-pass-filtered stethoscope signal using 25 ms frames with a 10 ms hop size using the mfcc function in MATLAB’s Audio Toolbox (Version 24.2, R2024b). Summary statistics (mean and standard deviation), as well as the first- and second-order derivatives, were then calculated for each MFCC. Additionally, motion features were derived from the IMU data by calculating the range of acceleration across the X, Y, and Z axes using both peak-to-peak and percentile-based measures.

Only participants for whom at least three valid breath cycles were successfully identified were retained for subsequent analysis. This criterion was established to maintain consistency with the guidelines from the ATS and European Respiratory Society (ERS) that recommend for a spirometry session to be considered valid, there must be at least three exhalation maneuvers performed [3]. As the number of successfully identified segments varied across participants and breathing conditions, some participants contributed data to both breathing conditions while others were exclusive to one, resulting in a partially crossed design across breathing modes.

### 2.8. Model Development

To reduce the dimensionality of the feature space while preserving variables with meaningful predictive value, preliminary feature selection was performed using the Boruta algorithm [30]. Boruta is a simulation-based framework built around the Random Forest classifier that evaluates feature relevance and robustness. The algorithm operates by generating “shadow features,” which are constructed by randomly shuffling the values of each predictor variable across observations over repeated iterations (e.g., 1000 iterations). This process yields a null distribution of feature importance against which observed feature importance is compared to determine statistical significance and reduce false discovery. Features that consistently outperform this null distribution across iterations are classified as important, while those that do not are rejected. This approach allows Boruta to retain all variables with statistically significant predictive signals rather than selecting only a minimal subset, making it particularly suitable for high-dimensional datasets.

In this study, the candidate feature set consisted of 185 potential predictors derived from tracheal sound and IMU signals. To account for potential differences in the information content of these modalities, the features were first grouped into tracheal-sound-derived and IMU-derived subsets, and the Boruta procedure was applied separately to each feature group and spirometric index to identify the most informative predictors for subsequent model development. It is important to note that because Boruta was applied to the full dataset prior to LOSO-CV iterations, this may introduce a degree of optimistic bias during preliminary feature selection. However, this selection is further refined during downstream model development, producing the final predictive feature sets based on model-specific performance and regularization.

After feature selection, the selected acoustic and IMU-derived variables were incorporated as candidate predictors for model development. These features were combined with participants’ biological characteristics (age, sex, BMI, and race/ethnicity), which were included as covariates in all models. Models were developed separately for each spirometric index, forced vital capacity (FVC), forced expiratory volume in one second (FEV_1_), and peak expiratory flow (PEF), and for each breathing condition (low-effort and forceful breathing), resulting in individual model pipelines per outcome and breathing mode. Predictive modeling was performed using both elastic net and simple linear regression approaches. The elastic net model was used to account for potential multicollinearity between sensor-derived feature groups, as Boruta was applied separately for each group and to perform coefficient shrinkage and additional variable selection through a combination of L1 and L2 regularization [31]. In parallel, linear regression models were implemented as a baseline modeling approach without regularization.

**Figure 3:**
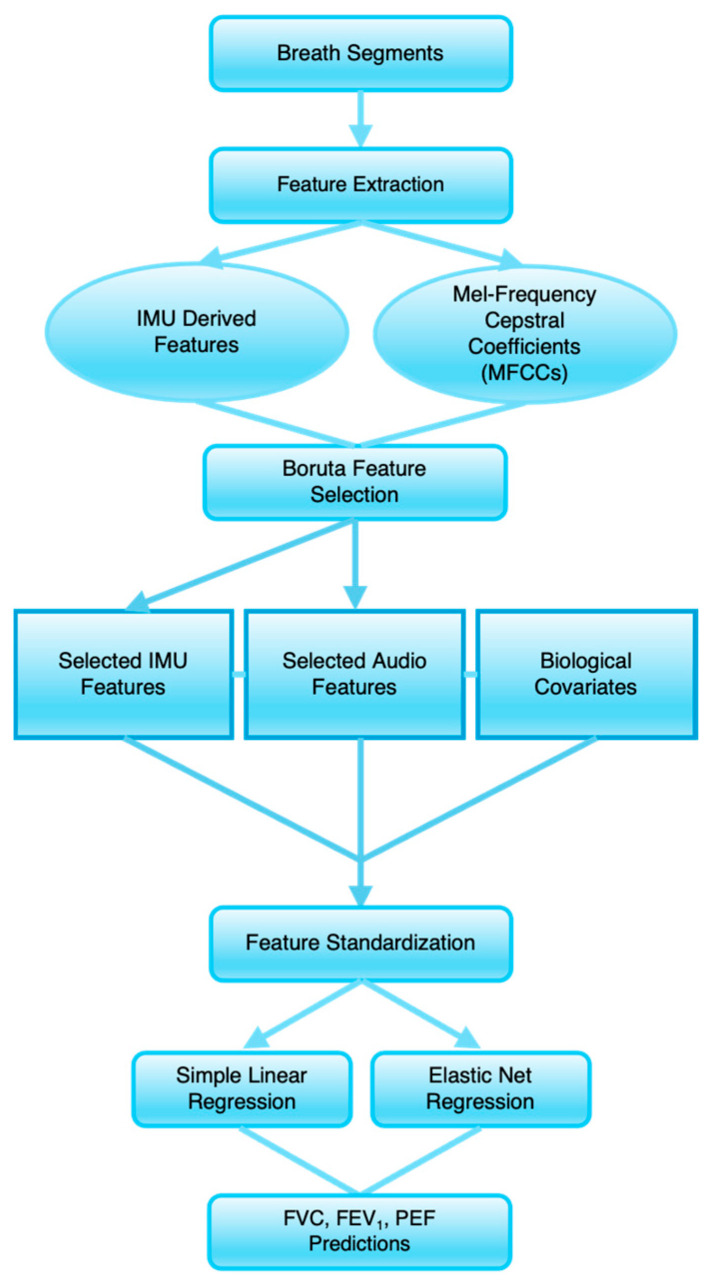
Overview of spirometric indices prediction.

Due to the limited sample size, a separate training and testing split was not performed. Instead, model performance was evaluated using both training error and leave-one-subject-out cross-validation (LOSO-CV) error to assess model fit and generalizability, respectively. For LOSO-CV, all observations from a given participant were iteratively held out as the validation set while the model was trained on the remaining subjects to prevent information leakage across folds. Predictive accuracy was assessed using root mean squared error (RMSE) and mean absolute error (MAE) calculated across the cross-validation folds. Hyperparameter tuning of the elastic net penalty (λ) and mixing parameter (α), as well as z-score standardization of predictors, were performed within each training fold of the LOSO-CV procedure. The best-performing models were then selected based on the lowest RMSE, with MAE as an additional criterion, reflecting the best overall predictive performance. To calculate training error, these models were then used to calculate predictions on the full datasets. Subject-level RMSE and MAE were computed and averaged across subjects, consistent with the subject-level aggregation used during LOSO-CV evaluation. The difference between training and LOSO-CV error indicates the degree of overfitting, with larger gaps suggesting poorer generalization to unseen subjects. The overall predictive modeling workflow is summarized in Figure 3.

To assess whether model prediction errors differed significantly between breathing modalities, separate linear mixed-effects models were fit for each spirometric index (FVC, FEV_1_, PEF) with subject-level RMSE as the outcome, breathing mode as a fixed effect, and a random intercept by participant. The random intercept specification represents an approximation of the true dependency structure, as the data were insufficient to support random slope estimation. Statistical significance was evaluated at an α level of 0.05.

## 3. Results and Discussion

### 3.1. Validation of DSP Performance

**Figure 4:**
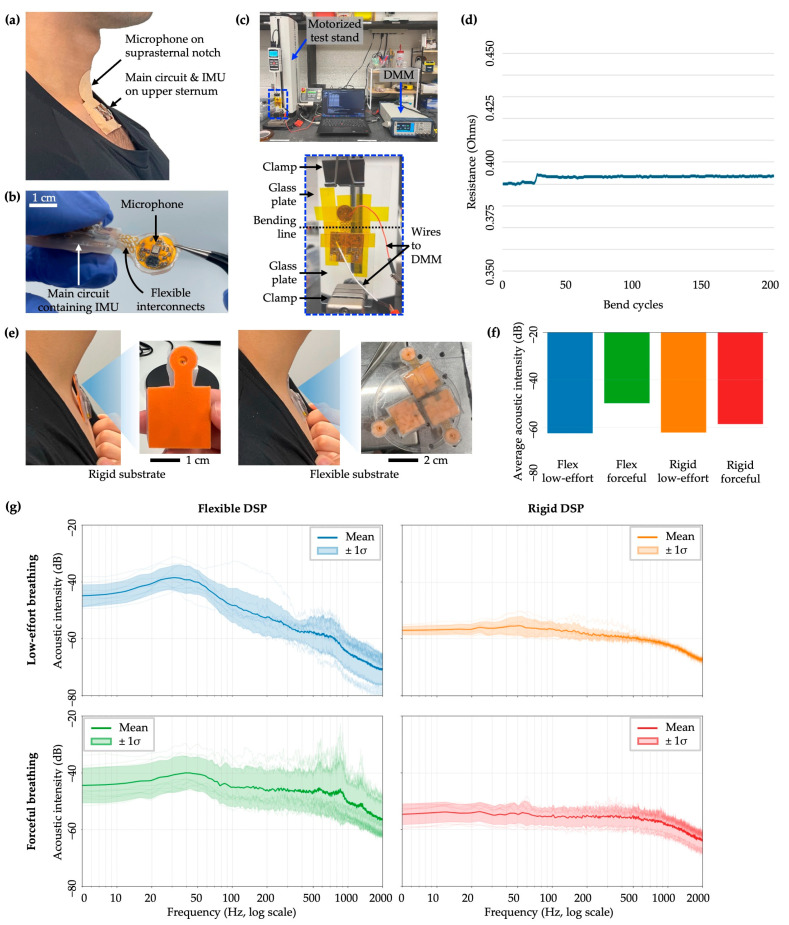
Validation of DSP performance. (**a**) Photograph showing the DSP applied over the sternal region, with arrows indicating the placement of the two functional components: the microphone island over the suprasternal notch and the main circuit containing the IMU over the upper sternum. (**b**) Photograph showing the mechanical flexibility enabled by the stretchable interconnect connecting the circular island containing the microphone chip to the main circuit. (**c**) Repeated bending test setup that includes the motorized stand and digital multimeter (DMM). The zoomed inset highlights the fPCB mounting configuration that enabled repeated bending while electrical resistance across the fPCB ground plane was continuously monitored. (**d**) Electrical resistance measurement data over 200 cycles of bending. (**e**) Preparation and application of the rigid and flexible DSPs for performance comparison. Zoomed-in panels show the backside configurations of the two form factors: a 1 mm thick PLA backing for the rigid DSP and a high- tack silicone gel backing (Silbione RT Gel 4717, Bluestar Silicones, East Brunswick, NJ, USA) for the flexible DSP. (**f**) Comparison of average acoustic intensity across the full spectrum for all participants under two breathing maneuvers (low-effort and forceful) and two device form factors (rigid and flexible). (**g**) Comparison of frequency-resolved acoustic intensity across two breathing maneuvers and two device form factors. The darker midline represents the mean across 9 total trials (3 trials from each of 3 individuals) and the lighter shaded region represents ±1 standard deviation around the mean.

The Digital Spirometry Patch (DSP) was developed as a skin-conformal wearable for simultaneous acquisition of tracheal sounds and chest motions at the lower anterior neck and upper sternal region. This platform builds on our previous patch-based respiratory sensing work reported by Lee et al. but extends that earlier design beyond adventitious sound detection. Whereas the prior system was used primarily for automatic detection of wheezes, crackles, and rhonchi, the present DSP was designed for multimodal sensing by integrating an MEMS microphone (ICS-40212, TDK InvenSense, San Jose, CA, USA) for tracheal acoustic capture and a three-axis MEMS accelerometer (ADXL355, Analog Devices, Wilmington, MA, USA) for chest motion sensing. The device’s layout was configured such that the circular microphone island was positioned at the suprasternal notch region where coupling to the trachea is strongest for airway sound capture, whereas the main circuit body was positioned more inferiorly over the upper sternum, where chest wall motion is more prominent (Figure 4a). These two components were connected by a serpentine stretchable interconnect, which mechanically decoupled the microphone island from the main circuit body and allowed each region to maintain conformal contact despite local deformation during breathing, swallowing, speaking, and routine neck movement. This flexible, body-conformal architecture was therefore important not only for wearability but also for preserving stable multimodal signal acquisition in an anatomically curved and mechanically dynamic region (Figure 4b). By combining these modalities within this mechanically adaptive design, the DSP was intended to capture complementary information related to airflow dynamics and thoracic mechanical displacement during exhalation.

As shown in Figure 4c, DSP’s mechanical robustness and electrical reliability were evaluated using repeated bending tests while monitoring electrical resistance across the serpentine interconnect. Bending was actuated using an ESM303 linear motorized test stand (Mark-10 Corporation, Copiague, NY, USA), which flexed the device to 80° for 200 cycles. This angle exceeded bending expected during routine wear and therefore served as a conservative test condition. Primary mechanical stress of this type is expected to occur during device removal rather than during normal wear. For the present study, each device was generally intended for use by a single participant over the study visit, corresponding to approximately one removal event per device, with occasional additional handling or reapplication events in some cases. Thus, the expected number of removal-associated bending events per device in the current use case was far below 200 cycles. More broadly, even under a future repeated-use scenario involving one application/removal event per day over multiple days, 200 cycles would correspond to approximately 200 days of use. Accordingly, the 200-cycle test was selected as a conservative benchmark relative to the intended use conditions evaluated in this study. For repeatable deformation, the flexible PCB was taped across a gap between two glass slides so that bending was localized to the serpentine region. Electrical continuity across the interconnect was monitored through jumper-wire connections to a digital multimeter, while both the stand and the meter were interfaced to a laptop for continuous recording. The measured resistance remained highly stable throughout testing, with a total increase of less than 5 mΩ across 200 cycles. The small step-like change observed during the test rapidly plateaued and did not continue to drift, suggesting that it was more likely attributable to environmental variation or contact effects than structural degradation of the interconnect (Figure 4d). These data indicate that the flexible serpentine connection tolerated repeated deformation without meaningful loss of electrical performance, supporting its suitability for use at the suprasternal notch.

We next performed a small pilot study to compare sensing performance between the flexible DSP and a rigid counterpart. The purpose of this experiment was to determine whether conformal coupling to the suprasternal notch improves capture of breath-related acoustic waveforms. To create the rigid condition, a 1 mm thick PLA backing was fabricated through 3D printing and attached beneath the device to simulate a rigid patch architecture (Figure 4e). Three healthy volunteers each completed three trials in both flexible and rigid configurations under two breathing conditions: low-effort breathing and forceful expiration. During low-effort breathing, participants inhaled deeply and exhaled in a sustained, non-forceful manner intended to approximate the low-effort maneuver targeted by the DSP. During forceful breathing, participants inhaled fully and then exhaled as rapidly and forcefully as possible to mimic conventional spirometric effort.

As shown in Figure 4f, when acoustic intensity was summarized as an average across the full spectrum, the flexible and rigid devices showed similar sound levels during low-effort breathing, whereas both configurations produced higher average intensity during forceful expiration. This pattern is physiologically expected because greater expiratory airflow generates stronger airway acoustic energy. At the same time, broadband signal level alone was not especially informative for the intended use case of this study. Forceful breathing maneuvers produce substantial turbulent airflow at the mouth, which may introduce non-target acoustic contributions, and the similarity in whole-spectrum averages between flexible and rigid devices during low-effort breathing suggests that aggregate sound level can obscure differences in physiologically relevant spectral content.

A clearer distinction emerged from the frequency-resolved analysis. In the low-effort breathing condition, the flexible device showed higher signal levels than the rigid device in the tracheal sound range, particularly around 800–1000 Hz. Because this band is closely associated with airflow-related tracheal acoustics, the result suggests that the flexible device provided more effective acoustic coupling to the suprasternal notch and better-preserved signal content relevant to respiratory assessment. The low-effort breathing data collected with the flexible device also showed a broader spread around the cohort-average curve than the rigid condition, consistent with preservation of physiologic and session-level variation in tracheal sound characteristics rather than attenuation caused by poor mechanical coupling. In contrast, the higher broadband intensity observed during forceful breathing should be interpreted cautiously, as turbulent airflow and sound radiated from the mouth and nostrils during expiration may contribute substantially to the recorded signal. Thus, greater acoustic intensity during forceful breathing does not necessarily indicate more specific or more informative tracheal sound capture.

Collectively, these findings support the flexible DSP architecture from both engineering and physiological perspectives. The device remained electrically stable under repeated bending conditions exceeding those expected during routine wear, and the flexible form factor improved capture of acoustically relevant tracheal signal content in the low-effort breathing condition most aligned with the intended clinical use scenario. These results therefore support the DSP as a mechanically robust and physiologically appropriate platform for wearable lung function assessment and provide the technical basis for subsequent prediction of spirometric parameters from multimodal patch data.

### 3.2. Breath Phase Detection Application

Part of the spirometric prediction pipeline involved identifying inspiration and expiration segments within each respiratory maneuver. The two phases of the respiratory cycle exhibit distinct acoustic and mechanical signatures associated with airflow and chest wall movement. Accurate segmentation is therefore essential to ensure that extracted features do not overlap across multiple respiratory phases, introducing signal contamination and potentially degrading model performance.

**Figure 5:**
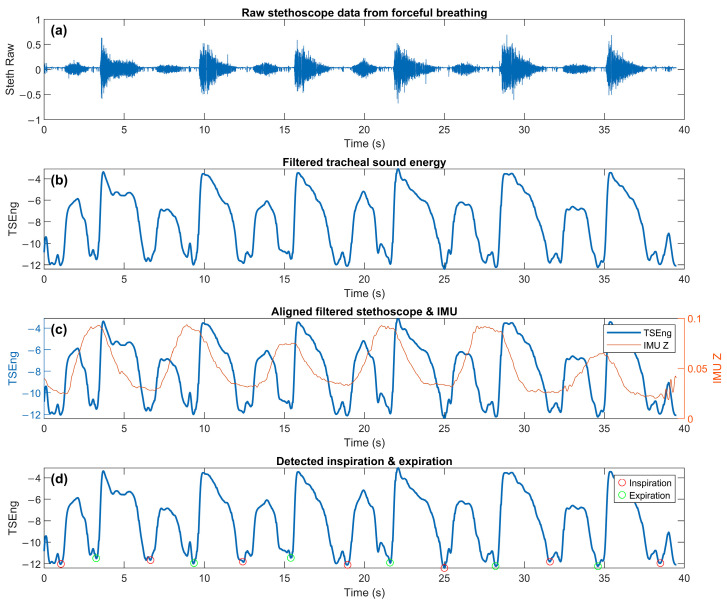
Respiratory phase detection algorithm. (**a**) Raw tracheal sound waveform captured during a forceful breathing maneuver. (**b**) Tracheal sound energy (TSEng), calculated as the log-variance of short-time-windowed, bandpass-filtered tracheal sound. (**c**) Overlay of filtered TSEng and filtered IMU Z-axis acceleration data. (**d**) Detected respiratory phases over time within tracheal sound energy, with inspiration (red) and expiration (green) markers.

Figure 5 demonstrates the application of our breath phase detection process using representative data from a forceful breathing sequence. The raw tracheal stethoscope signal (Figure 5a) exhibits high-frequency acoustic fluctuations associated with turbulent airflow. After processing, the resulting tracheal sound energy envelope provides a clearer representation of the respiratory pattern (Figure 5b), allowing individual breathing cycles to be more readily identified. Alignment of the acoustic signal with the Z-axis accelerometer signal demonstrates the relationship between airflow-related acoustic energy and thoracic motion captured during breathing (Figure 5c). Using these complementary signals, inspiration and expiration events were identified and used to segment the respiratory signal into discrete phases to be used for subsequent feature extraction and downstream analysis (Figure 5d).

Using the breath phase detection algorithm, valid respiratory cycles were identified for a subset of participants in each breathing condition. From the full cohort of 18 participants, 9 provided sufficient data for low-effort breathing analysis and 14 for forceful breathing analysis, defined as having at least three valid inspiration–expiration segment pairs identified. While low-effort breathing reduces user burden, it also introduced the most challenges in signal segmentation. The smaller number of usable recordings during the low-effort breathing condition is likely attributed to the lower signal intensity associated with less forceful respiratory maneuvers. Low-effort breathing produces weaker airflow and reduced thoracic motion compared with forceful exhalation, which may result in lower-amplitude acoustic and accelerometer signals. As a result, respiratory events may be less distinct, making phase boundaries more difficult to detect. Consequently, this reduced the amount of useable training data available for low-effort breathing models and can negatively impact predictive performance and generalizability. In contrast, forceful breathing generates stronger turbulent airflow and larger chest wall movement, producing clearer acoustic and motion signatures that facilitate more adequate phase identification.

### 3.3. Model Performance

Both standard linear regression and elastic net models were developed to predict spirometric indices, forced vital capacity (FVC), forced expiratory volume in one second (FEV_1_), and peak expiratory flow (PEF), from patch-derived features alongside participant demographic covariates. Final predictive performance was evaluated using leave-one-subject-out cross-validation (LOSO-CV), with 9 participants included for low-effort breathing and 14 for forceful breathing. Linear regression served as a baseline, non-regularized approach but demonstrated consistently lower predictive performance, with elastic net improving LOSO-CV RMSE by 44.4% and 80.7% on average across spirometric indices for forceful and low-effort breathing, respectively. This likely reflects instability in coefficient estimation in the presence of correlated and high-dimensional predictors. Sensor-derived acoustic and IMU features are often highly collinear, which can inflate coefficient variance and reduce generalization in simple ordinary least squares models. Elastic net regularization addresses this through simultaneous coefficient shrinkage and variable selection via combined L1 and L2 penalties, stabilizing estimates in the presence of correlated predictors and resulting in more robust predictive performance. Subsequent evaluation therefore focused on elastic net models. Across these models, we observed that the inclusion of biological demographic covariates produced non-zero coefficients in the majority of cross-validation folds, suggesting a recurrent contribution to model predictions. However, given the small sample size and the number of models compared across breathing conditions and spirometric indices, the relative contribution to predictive performance of patch-derived signals versus demographic covariates cannot be conclusively partitioned at this stage and the subsequent findings are better interpreted as preliminary evidence of the feasibility of this multimodal framework.

**Figure 6:**
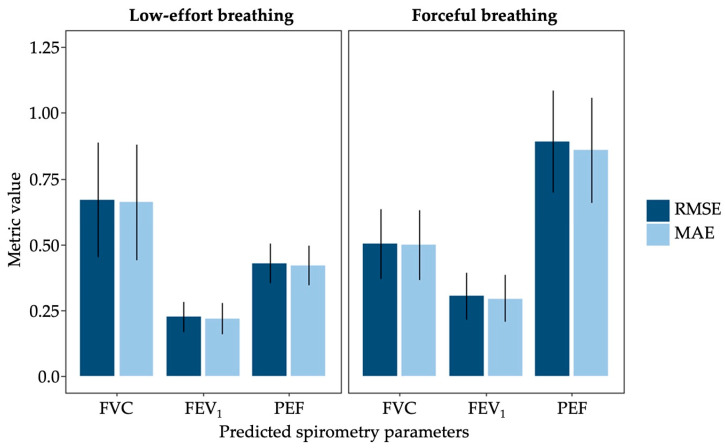
One-subject-out cross-validation performance of elastic net regression models across breathing modes. Root mean squared error (RMSE, dark blue) and mean absolute error (MAE, light blue) for models predicting spirometry indices (FVC, FEV1, and PEF) under forceful and low-effort breathing conditions. Error bars represent standard error of the mean.

The ATS/ERS repeatability criteria require that out of three maneuvers, the difference between the two largest FVC values, as well as the two largest FEV_1_ values, be less than 0.15 L [3]. Earlier guidelines recommended that the two highest recorded PEF values should differ by no more than 0.67 L/s [32]. As these thresholds reflect the magnitude of acceptable within-subject measurement variability and given that the LOSO-CV procedure evaluates model performance across repeated breaths within each held-out subject, we used these values as performance benchmarks. Accordingly, our goal was to achieve RMSE values at or below these thresholds for each respective spirometric index. The elastic net models demonstrated varying training and predictive performance across spirometric indices and breathing conditions, as summarized in Figure 6. For low-effort breathing maneuvers (*n* = 9), the best-performing models achieved training RMSE values of 0.315 L for FVC, 0.091 L for FEV_1_, and 0.149 L/s for PEF, with corresponding LOSO-CV RMSE values of 0.668 L, 0.224 L, and 0.428 L/s, respectively. For forceful breathing maneuvers (*n* = 14), the training RMSE values were 0.314 L for FVC, 0.141 L for FEV_1_, and 0.649 L/s for PEF, with corresponding LOSO-CV RMSE values of 0.499 L, 0.304 L, and 0.891 L/s respectively. The modest gap between training and LOSO-CV error across both breathing conditions indicates limited overfitting, suggesting that elastic net regularization may help constrain model complexity despite the small sample sizes. In addition, linear mixed-effects models found no significant association between breathing mode and subject-level RMSE for FVC (*p* = 0.737), FEV_1_ (*p* = 0.520), and PEF (*p* = 0.073). Only the model predicting PEF using low-effort breathing achieved the targeted within-subject measurement variability, whereas the other models’ errors were greater than the accepted difference. However, the results indicate that the combination of wearable patch-derived features and demographic covariates was still able to capture information relevant to spirometric lung function measures.

Although no statistically significant differences in model performance were observed across breathing modes, the observed differences in predictive performance across breathing modalities may reflect the physiological characteristics captured by each maneuver. Forceful breathing more closely resembles the standard spirometry maneuver used to measure FVC, which may explain the improved prediction of FVC during forceful breathing. Notably, low-effort breathing produced the only model that met our reproducibility criteria for PEF, suggesting that the more controlled and repeatable airflow patterns during low-effort breathing may allow for more stable model prediction, however, this should be interpreted cautiously alongside the observed breath phase segmentation failures. Collectively, these findings indicate that acoustic and thoracic motion signals acquired by the DSP during low-effort breathing can support spirometric prediction, underscoring the device’s potential as a passive, accessible alternative to traditional pulmonary function testing. At the same time, it is important to note that the presented study was conducted exclusively in healthy adult volunteers, and the cohort was relatively small and demographically homogeneous. Although this design is appropriate for early-stage device validation, where healthy participants are commonly used to establish technical feasibility and baseline performance under controlled conditions, it does not reflect the clinical population for whom the DSP is ultimately intended. Patients with asthma, COPD, and other respiratory disorders exhibit altered lung mechanics, reduced expiratory flow, and greater physiological heterogeneity, each of which may affect the acoustic and motion signals captured by the patch as well as the accuracy of the derived predictions. Accordingly, the authors intend to assess DSP performance in a larger and more clinically representative cohort as part of the development and clinical evaluation pipeline following this pilot study.

## 4. Conclusions

In this study, we presented the Digital Spirometry Patch (DSP), a soft, skin-conformal wearable device designed to estimate clinically standard spirometric indices through low-effort breathing maneuvers. Unlike traditional spirometry, which depends on maximal patient effort and technician supervision, the DSP leverages multimodal tracheal acoustic and chest motion signals to capture physiologically relevant respiratory features during low-effort breathing without the need for additional accessories such as mouthpieces and nose clips. Using elastic net regression evaluated by leave-one-subject-out cross-validation, the DSP demonstrated the potential to estimate FVC, FEV_1_, and PEF from patch-derived features combined with demographic covariates. These findings provide evidence for proof of concept that low-effort, wearable-based lung function assessment is feasible and that the combination of tracheal sound, thoracic motion signals, and demographic covariates acquired through the DSP framework carries sufficient information to support spirometric prediction. The results also represent a meaningful progression from our previous work focused on qualitative respiratory sound detection to objective estimation of spirometric indices.

The modeling approach utilized in the project was based on unregularized and regularized linear methods. Although elastic net partially accounts for multicollinearity and improves generalization through regularization, it still assumes predominantly linear relationships between patch input features and spirometric outcomes. Our analysis suggested that simple ordinary least squares could not capture the complex physiological relationships between tracheal acoustics, chest wall motion, and pulmonary function. Relatedly, the reduced number of valid respiratory segments obtained during low-effort breathing highlights a limitation in signal detectability during low-effort maneuvers. The lower airflow and reduced thoracic motion characteristics of low-effort breathing produce weaker acoustic and accelerometer signals, making respiratory phase boundaries more difficult to detect reliably. The combination of these factors reduced the amount of useable training data and possibly worsened predictive performance for low-effort breathing. Future work should therefore explore deep learning architectures, such as convolutional neural networks (CNNs), recurrent neural networks (RNNs), or transformers, which may simultaneously help address these limitations. Such architectures are capable of capturing nonlinear, high-dimensional relationships that are characteristic of multimodal sensor signals and spirometric outcomes without relying on assumptions of linearity. These methods could improve the identification of subtle respiratory phase transitions during low-effort breathing maneuvers.

Although signal acquisition and visualization were performed through the wearable platform and the connected mobile device, the prediction pipeline in this study was conducted offline. In the next stage of development, we will integrate this prediction framework directly into the mobile application to enable real-time or near-real-time lung function estimation during use. In addition, because the present study was conducted under controlled experimental conditions, future work should expand data collection to naturalistic home and daily-life environments, where posture, ambient noise, and user behavior may differ substantially from the laboratory setting. Incorporating such real-world data will be important for improving model robustness and enabling prediction under the conditions in which remote respiratory monitoring would ultimately be used. Finally, the relatively small sample size of this proof-of-concept study (*n* = 14 for forceful breathing, *n* = 9 for low-effort breathing) required model evaluation using leave-one-subject-out cross-validation rather than training and testing on fully independent cohorts. These findings cannot capture the full range of physiological variability, lung function severity, and demographic diversity that would exist in a broader clinical population and, as such, the findings should be interpreted as an early technical proof of concept establishing a performance baseline in a controlled healthy cohort consistent with established practice in early stage medical device development, rather than a definitive clinical evaluation. A larger follow-up study with broader participant diversity will allow for more rigorous model development and validation, including stronger external evaluation of generalizability across users and settings. Formal partitioning of the relative contributions of patch-derived signals versus demographic features is also identified as an important direction for evaluating the patch. Taken together, these next steps will help establish the DSP as a practical and scalable platform for accessible pulmonary monitoring outside of conventional clinical settings, with potential value for longitudinal disease management and remote patient care.

## Figures and Tables

**Table 1:** Comparison of DSP to existing systems and work (‘—’ indicates information not reported in the published literature).

Form Factor	Reference Devices/Work	Required Breathing Maneuver	Utilized Sensor Data	Generated Pulmonary Data	Evaluation Method	Reported Accuracy	Requires Additional Accessories?
Handheld spirometers	MIR Spirobank Smart	Maximal effort expiration	Air flow	FVC, FEV_1_, PEF, and more	—	≤6% predicted difference vs lab spirometry	Yes (nose clip, mouthpiece)
Smartphone microphone	SpiroCall	Maximal effort expiration	Exhalation sound	FVC, FEV_1_, PEF, FEV_1_%	Cross-validation	Mean error of 7.2% across spirometric indices	Yes (mouthpiece)
	SpiroSmart	Maximal effort expiration	Exhalation sound	FVC, FEV_1_, PEF, FEV_1_%	Multi-split, ensemble aggregation	Mean error of 5.1% across spirometric indices	Yes (mouthpiece)
	SpiroSonic	Maximal effort expiration	Exhalation sound, chest wall motion	FVC, FEV_1_, PEF, FEV_1_%	Cross-validation	Mean error of 2.5% across spirometric indices	No
	Sharan et al.	Cough	Cough sound	FVC, FEV_1_, FEV_1_%	Train–test	RMSE of 0.593 L, 0.725 L, and 0.164 for FVC, FEV_1_, and FEV_1_%, respectively	No
External microphone	Alam et al.	Speech	Speech sound	FEV_1_%	Cross-validation	RMSE of 11.47 and MAE of 8.96 for FEV_1_%,	No
Chest strap	Resmetrix	Normal breathing	—	Respiratory pattern	—	—	No
Wearable patch	Strados Lab, ADAMM-RSM-SM	Normal breathing	Chest sound and movement	Adventitious sound detection	—	—	No
	Soft wearable stethoscope patch	Normal breathing	Chest sound	Adventitious sound detection	—	—	No
	Digital Spirometry Patch (this work)	Low-effort breathing	Tracheal sound, chest wall motion	FVC, FEV_1_, PEF	Cross-validation	RMSE of 0.668 L, 0.224 L, and 0.428 L/s for FVC, FEV_1_, and PEF, respectively	No

**Table 2:** Participant characteristics.

Participant Characteristics (*N* = 18)	
Age (years)	30.3 ± 6.7
Male (*n*, %)	8 (44.4%)
Ethnicity (*n*, %)	
Asian	6 (33.3)
Caucasian	8 (44.4)
Southeast Asian	1 (5.6)
Other	3 (16.7)
BMI (kg/m^2^)	23.9 ± 3.3
FVC (L)	4.0 ± 1.1
FEV_1_ (L)	3.2 ± 0.7
PEF (L/s)	6.9 ± 1.7

Numeric variables are reported as mean ± SD.

## Data Availability

The original contributions presented in this study are included in the article/Supplementary Materials. Further inquiries can be directed to the corresponding authors.

## Supplementary Materials

The following supporting information can be downloaded at: https://www.mdpi.com/article/10.3390/bios16050272/s1, Table S1: End of session survey questions by category. Participants responded using a 5-point Likert scale (0–4) to each question, ranging from Strongly Disagree to Strongly Agree; Figure S1: Block diagram of the key electronic components of the Digital Spirometry Patch and downstream utilization of sensor data; Figure S2: Rank distribution of participant survey responses.
