# Supplementary material for: Low-Effort Respiratory Function Estimation with a Soft Wearable Digital Spirometry Patch
Source: Biosensors (Basel). 2026 May 8;16(5):272. doi: 10.3390/bios16050272 (PMC13204607; doi:10.3390/bios16050272)
Supplement: Supplementary file 1 [file biosensors-16-00272-s001.zip › biosensors-4250577-supplementary.pdf]

biosensors

MDPI

Article

Supplementary Information for **Low-Effort Respiratory Function Estimation with a Soft Wearable Digital Spirometry Patch**

Faheem A. Karim [1,2,†,‡], Ahmed Tariq [1,2,‡], Christopher B. Fitzpatrick [3], Lauren Zhou [4], Mayte Suárez-Fariñas [1,5,6], Helena Schotland [1], Linda Rogers [1], Yoon Jae Lee [2,7], Woon-Hong Yeo [4,8,9,10,*] and Yun Soung Kim [1,2,11,*]

[1] Icahn School of Medicine at Mount Sinai, New York, NY 10029, USA; faheem.karim@nyu.edu (F.A.K.); ahmed.tariq@icahn.mssm.edu (A.T.); mayte.suarezfarinas@mssm.edu (M.S.-F.); helena.schotland@mssm.edu (H.S.); linda.rogers@mssm.edu (L.R.)
[2] BioMedical Engineering and Imaging Institute, Icahn School of Medicine at Mount Sinai, New York, NY 10029, USA
[3] School of Electrical and Computer Engineering, Georgia Institute of Technology, Atlanta, GA 30332, USA; cfitzpatrick7@gatech.edu (C.B.F.)
[4] George W. Woodruff School of Mechanical Engineering, Georgia Institute of Technology, Atlanta, GA 30332, USA; laurenzhou@gatech.edu (L.Z.)
[5] Center for Biostatistics, Department of Population Health Science and Policy, Icahn School of Medicine at Mount Sinai, New York, NY 10029, USA
[6] Icahn Institute for Data Science and Genomic Technology, Icahn School of Medicine at Mount Sinai, New York City, NY 10029, USA
[7] Department of Computer Science, Georgia State University, Atlanta, GA 30303, USA; yoonlee@gsu.edu (Y.J.L.)
[8] Wallace H. Coulter Department of Biomedical Engineering, Georgia Tech and Emory University, Atlanta, GA 30332, USA
[9] Wearable Intelligent Systems and Healthcare Center (WISH Center), Institute for Matter and Systems, Georgia Institute of Technology, Atlanta, GA 30332, USA
[10] Atlanta, GA 30332, USA Parker H. Petit Institute for Bioengineering and Biosciences, Georgia Institute of Technology
[11] Department of Diagnostic, Molecular, and Interventional Radiology, Icahn School of Medicine at Mount Sinai, New York, NY 10029, USA
* Correspondence: whyeo@gatech.edu (W.-H.Y.); yunsoung.kim@mssm.edu (Y.S.K.)
† Current Address: Translational Research Center, Department of Oral & Maxillofacial Surgery, New York University College of Dentistry, New York, NY 10010, USA.
‡ These authors contributed equally to this work.

Table S1. End of session survey questions by category. Participants responded using a 5-point Likert scale (0–4) to each question, ranging from Strongly Disagree to Strongly Agree.

Category	ID	Question
Benefit	Q1	I can benefit from this technology
	Q2	The effort of using this technology is worthwhile for me
	Q3	I would recommend this technology/method to other people
Usability	Q4	I am satisfied with how easy it is to use this device
	Q5	The use of these patches requires a great deal of effort
	Q6	I feel safe when using the digital stethoscope patches
	Q7	I would feel safe using these patches daily if I had to
Wearing Comfort	Q8	Wearing the digital stethoscope patches is comfortable
	Q9	The digital stethoscope patches are comfortable to apply to the skin
	Q10	The digital stethoscope patches are comfortable to remove from the skin
	Q11	I am pleased with the size of the patches
	Q12	I would like a different design of the patches
Future Use	Q13	I can see myself using these patches for personal monitoring of my respiratory health
	Q14	If these patches could sync with my smartphone or health app, I would be more likely to use it regularly
	Q15	I am interested in receiving feedback or updates from the device about my respiratory health
	Q16	I can comfortably wear the patches continuously for one week
	Q17	I can comfortably wear the patches continuously for two weeks
	Q18	I can comfortably wear the patches continuously for one month

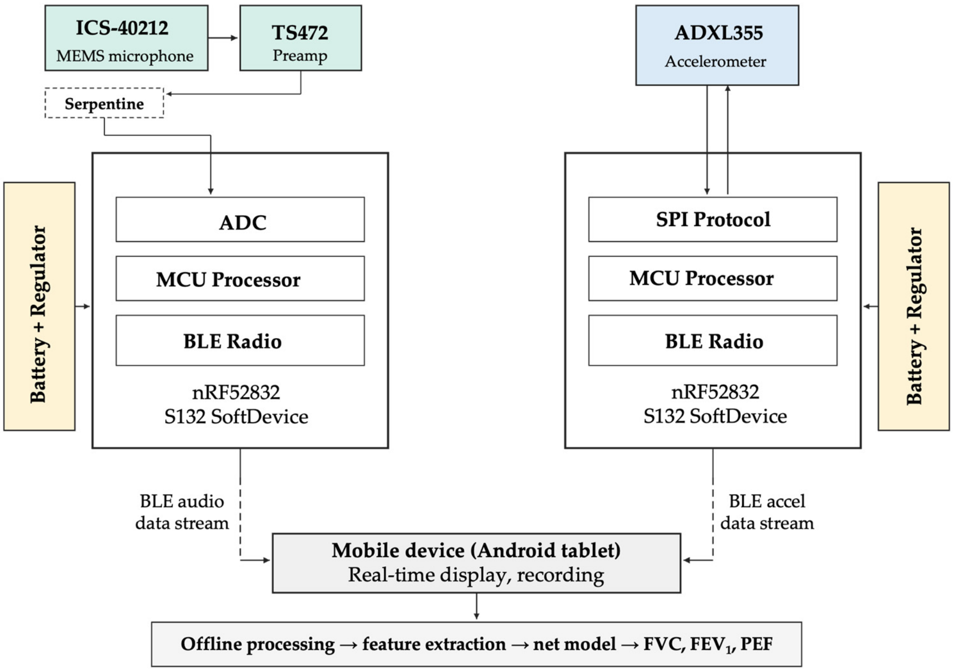

Figure S1. Block diagram of the key electronic components of the Digital Spirometry Patch and the downstream utilization of sensor data.

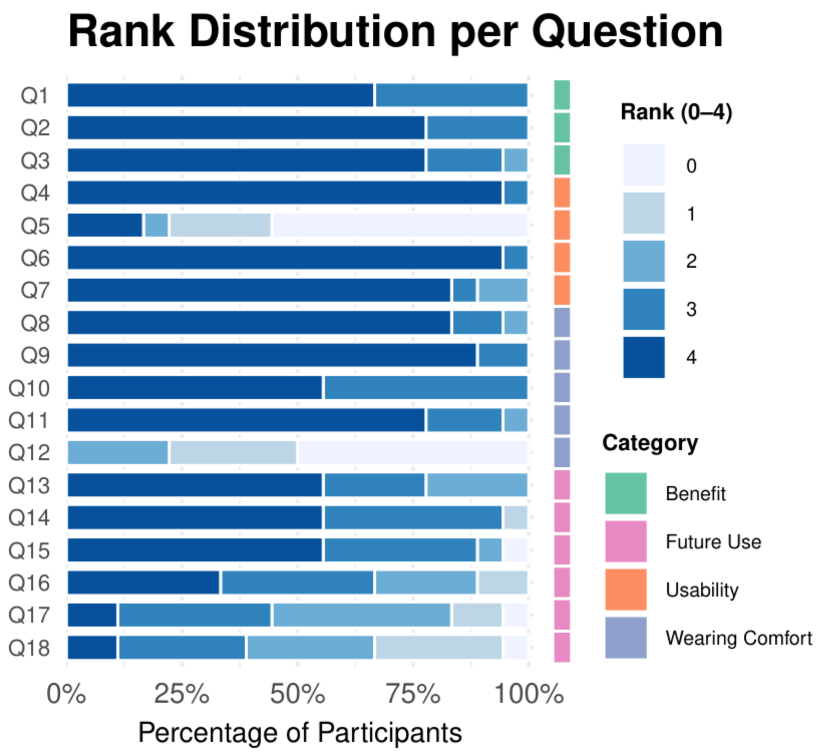

Figure S2. Rank distribution of participant survey responses.
